# Precise control of water stress in the field reveals different response thresholds for forage yield and digestibility of maize hybrids

**DOI:** 10.3389/fpls.2023.1142462

**Published:** 2023-03-14

**Authors:** Oscar Main, Marie-Pierre Jacquemot, Yves Griveau, Sophie Guillaume, Claire Demonceaux, Paul-Louis Lopez-Marnet, Sébastien Rey, Sébastien Fargier, Pascal Sartre, Christophe Montagnier, Anthony Uijttewaal, Nathalie Mangel, Florence Meunier, Matthieu Reymond, Valérie Méchin, Sylvie Coursol

**Affiliations:** ^1^ Université Paris-Saclay, INRAE, AgroParisTech, Institut Jean-Pierre Bourgin (IJPB), Versailles, France; ^2^ Unité Expérimentale DiaScope, INRAE, Mauguio, France; ^3^ Unité Expérimentale Versailles-Saclay, INRAE, Versailles, France; ^4^ ARVALIS – Institut du végétal, Station expérimentale de La Jaillière, Loireauxence, France; ^5^ ARVALIS – Institut du végétal, Station expérimentale, Boigneville, France

**Keywords:** forage maize, water stress, yield, resilience, digestibility, NIRS, lignin content

## Abstract

**Introduction:**

With dwindling global freshwater supplies and increasing water stress, agriculture is coming under increasing pressure to reduce water use. Plant breeding requires high analytical capabilities. For this reason, near-infrared spectroscopy (NIRS) has been used to develop prediction equations for whole-plant samples, particularly for predicting dry matter digestibility, which has a major impact on the energy value of forage maize hybrids and is required for inclusion in the official French catalogue. Although the historical NIRS equations have long been used routinely in seed company breeding programmes, they do not predict all variables with the same accuracy. In addition, little is known about how accurate their predictions are under different water stress-environments.

**Methods:**

Here, we examined the effects of water stress and stress intensity on agronomic, biochemical, and NIRS predictive values in a set of 13 modern S0-S1 forage maize hybrids under four different environmental conditions resulting from the combination of a northern and southern location and two monitored water stress levels in the south.

**Results:**

First, we compared the reliability of NIRS predictions for basic forage quality traits obtained using the historical NIRS predictive equations and the new equations we recently developed. We found that NIRS predicted values were affected to varying degrees by environmental conditions. We also showed that forage yield gradually decreased as a function of water stress, whereas both dry matter and cell wall digestibilities increased regardless of the intensity of water stress, with variability among the tested varieties decreasing under the most stressed conditions.

**Discussion:**

By combining forage yield and dry matter digestibility, we were able to quantify digestible yield and identify varieties with different strategies for coping with water stress, raising the exciting possibility that important potential selection targets still exist. Finally, from a farmer’s perspective, we were able to show that late silage harvest has no effect on dry matter digestibility and that moderate water stress does not necessarily result in a loss of digestible yield.

## 1 Introduction

French selection criteria for forage maize in the second half of the 20th century were mainly centred on agronomic performance, including yield, precocity, disease and lodging resistance. The adoption of earlier-flowering hybrids, particularly well-suited to the Northern milk-production regions, increased cultivation surfaces from 350,000 ha in the 1970s to over 1.5 million ha in 2016. Over the same period, whole plant yield increased from 6 tons of dry matter (DM)/ha to over 20 tons DM/ha thanks to genetic progress (Baldy et al., 2017). These criteria were officialised in 1986 by the opening of a “forage maize” section in the official French maize hybrid registration catalogue (Surault et al., 2005).

The primary use of forage maize remains that of being the main element of animal feed. This capacity is quantified *via* a translation into energetic values. Net energy is traditionally expressed in France *via* a “barley feed unit” system wherein one kilogram of barley fed to the animal is equivalent to one Unit of Feed representing 1,760 kcal (“Unité Fourragère”, UF) (Vermorel et al., 1987). For milk production, we talk about “Unité Fourragère Laitière” (UFL). The first prediction models for UFL values were established by Andrieu, 1995. They consisted of a set of four different models, of which the M4 model was retained as the most accurate. This model was updated by Peyrat et al., 2016 to the M4.2 model, which remains the standard industry-wide model to date. This equation consists of two main variables, protein content (MAT) in g/kg of organic matter (OM) and DM digestibility in percentage of DM:


UFL (per 100 kg organic matter) = 18.77 + 0.1389*MAT + 0.9491*DM digestibility

According to the equation, the UFL value is much more dependent on the DM digestibility value than on MAT. Relations between UFL and DM digestibility, therefore, are almost always highly correlated (Baldy et al., 2017), and the low variability of the MAT value in modern hybrids often results in the equation being predicted using only the DM digestibility value.

Modern forage maize hybrids typically vary between a UFL value of about 0.86 and 0.92 (Baldy et al., 2017), a small variation that can have a significant impact on milk production, as a 0.05-point variation in UFL represents around 1.8 kg of milk per cow per day (Barrière, 2000). Although forage digestibility (and, by consequence, UFL value) was established in the 1980s, little work was done to incorporate it into selection. Between 1985 and 2000, genetic progress resulted in significant productivity increases and resistance to fall, probably due to an increased priority on grain value. During the same period, registered hybrids were on average 0.05 UFL points weaker than previously registered varieties (Barrière, 2000). A large number of studies have shown that DM digestibility, and therefore UFL, is controlled by cell wall (CW) content on the one hand and its digestibility on the other. Thus, CW digestibility (often measured as *in vitro* digestibility of CW residue, hereafter referred to as CW digestibility) is highly correlated to DM digestibility and UFL (Baldy et al., 2017). CW of modern hybrids was found to be 5.5 points less digestible compared to hybrids from the 1950s, resulting in a 2-point reduction in OM digestibility. This finding coincided with a 5-point increase in DM yield over the same period (Barrière et al., 2004). In 1998, to counteract this continuous decline in forage quality, the UFL value was included as an acceptance criterion to the official forage maize hybrid catalogue, replacing the minimum grain yield criterion (Perspectives Agricoles, n° 330). The impact of these new mandatory criteria was not immediate: the UFL value of new hybrids continued to decline until 2007 and then slowly increased until 2014-2015 (Surault et al., 2005; Baldy et al., 2017).

The new high-throughput *in vitro* methods (Lopez-Marnet et al., 2021), although far simpler and more efficient than the original *in vivo* digestibility methods, are still considered insufficient to cope with the large number of samples and limited time schedule required to predict UFL values in the modern hybrid selection procedure. Near-infrared spectroscopy (NIRS) has long been favoured in the agricultural sector because of its ability to determine a wide range of parameters. Their use in the forage maize market first began in the Americas (Norris et al., 1976; Valdes et al., 1987), using the equation of Norris et al., 1976, with specific attention to the CW-related parameters. For the French market, developments with predictive equations for OM digestibility started at the “Centre wallon de Recherches agronomiques” (CRA-W) in Gembloux in collaboration with the French seed company Limagrain (Biston and Dardenne, 1985). This first equation showed relatively good correlation values (r^2^ = 0.6; Biston et al., 1989) after calibration on caged-sheep experiments. The current industry-standard CRA-W Gembloux equations consist of thousands of data points that are intended to represent a wide range of genetic and environmental diversity. However, the current equations do not accurately predict all variables with the same level of precision (Mentink et al., 2006). In general, DM constitution and parameters are accurately predicted (DM digestibility r^2^ = 0.9; Andueza et al., 2011) and with low error rates. Difficulties were particularly noted in predicting neutral-detergent fiber (NDF) and its digestibility (Mentink et al., 2006; Bastianelli et al., 2019). This is particularly problematic for selection programs: as previously shown, these parameters influence OM digestibility and overall quality of forage maize, but are insufficiently considered in selection programs partly for this reason (Barrière et al., 2004).

At last, these considerations, whether they relate to agronomic traits such as yield or to silage quality traits such as DM digestibility, must be placed in the current context of climate change. Indeed, future climate projections indicate a substantial increase in the frequency and intensity of drought events (Lemaire, 2008; Harrison et al., 2014). Even if the 2°C warming target set by COP26 were met, a large proportion of French forage maize growing areas would suffer from drought (Roudier et al., 2016). Forage maize is particularly sensitive to water stress during the female flowering stage (Bänziger et al., 2000) which occurs in mid-July, and is therefore at risk of yield loss during this period (Salter and Goode, 1967). With the world’s dwindling freshwater supplies and increasing occurrences of water stress, agriculture is rapidly coming under increasing pressure to reduce water consumption while maximising use efficiency (Tester and Langridge, 2010; Messina et al., 2020). While the effects of such stresses on yield and plant health are well studied (Harrison et al., 2014; van der Velde et al., 2012), little is known about how water stress affects energy values of forage maize hybrids and how efficient biochemical and NIRS predictive values are under these highly contrasting conditions.

Here, we investigated the effects of water stress and stress intensity on agronomic, biochemical, and NIRS predictive values in a set of 13 forage maize varieties representative of the French S0-S1 market grown under four different environmental conditions resulting from the combination of a northern and a southern location and two monitored water stress levels in the south. We showed that agronomic performance gradually decreased as a function of water stress, while both DM and CW digestibilities increased regardless of the intensity of water stress, with variability among the tested varieties decreasing under the most stressed conditions. We also showed that the NIRS-predicted values were affected to varying degrees by environmental conditions. Finally, by combining yield and DM digestibility, we were able to quantify digestible yield and identify varieties with different strategies for coping with water stress.

## 2 Materials and methods

### 2.1 Hybrid material and field trials

Thirteen early-flowering forage maize hybrids (S0 and S1 earliness groups) were selected from the 2021 ARVALIS - Institut du végétal Post Inscription Evaluation Network to maximise variability between yield and UFL, while representing the French forage market through eight different breeding companies (**Supplementary Table 1**).

The hybrids were grown in two INRAE experimental units (EU) in France during the 2021 growing season: Versailles-Saclay EU (Versailles; GPS, N: 48’48”33.391/E: 2’5”7.237) and DiaScope EU (Mauguio; GPS, N: 43’36”52.438/E: 3’58”34.419), hereafter referred as “Versailles” and “Mauguio” for simplicity. While Mauguio is not a typical forage maize growing area, the Mediterranean climate at this location increases the probability of natural water deficits. Fields were sown at Versailles on April 28 and at Mauguio on May 7. Each line was grown in two lines and a planting density of 100,000 plants/ha. A total of five growing conditions were established at both sites:

- Two harvest stages in Versailles with irrigation by rainfall only, the amount of which could be monitored by a nearby climatic station and was sufficient during the flowering period (**Supplementary Figure 1**): a regular harvest with 32% DM content (RW) and a late second harvest with 40% DM (RW.40).
- Three different irrigation conditions in Mauguio piloted thanks to the use of tensiometers: a well-watered condition (WW) with ramp irrigation three times a week with 20 mm of irrigation, a moderate water deficit condition (WD1) with ramp irrigation of 15 mm when hydric tension reaches –125 kPa, a severe water deficit condition (WD) with ramp irrigation of 13 mm when hydric tension reaches –300 kPa.

**Figure 1:**
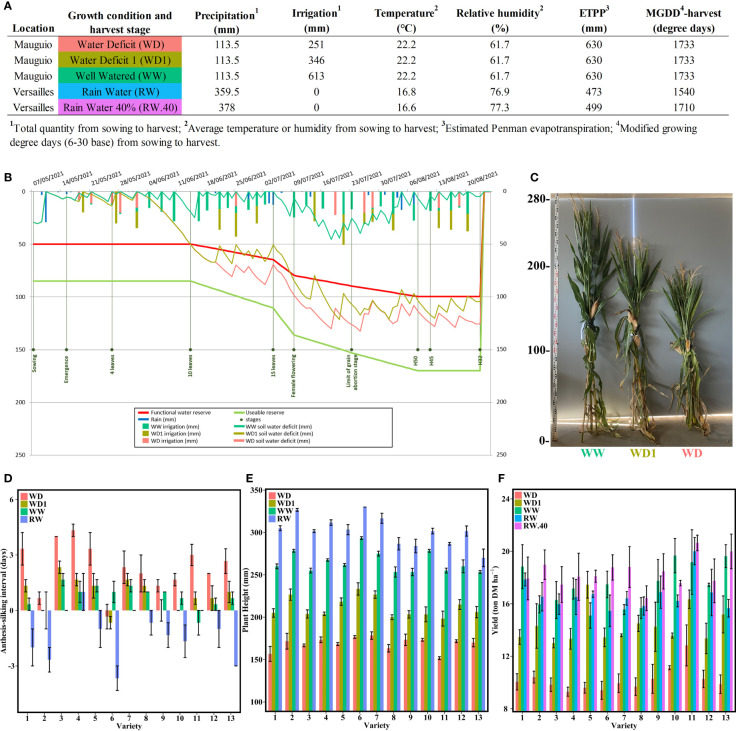
Harvest conditions and resulting effects on key agronomic parameters. **(A)** Main climatic and maintenance data related to the five different conditions studied. **(B)** Water balance sheet generated by the Irrélis irrigation advice software (Arvalis – Institut du vegetal) for the Mauguio field trials. The data shown were measured on variety 8 in the WW condition. **(C)** Representative photo of the resulting plant heights for the variety 4 under the three conditions at Mauguio. **(D)** Anthesis-silking interval for each variety in Mauguio and Versailles. **(E)** Mean plant height in cm at harvest from the four conditions at Mauguio and Versailles. **(F)** Mean yield in tons of DM per ha for all varieties under the five conditions in Mauguio and Versailles. The bars represent the means and the error bars indicate the standard error margins.

When conditions are grouped in the following text, the two-water deficit (WD and WD1) conditions are referred to as “dry” and the three rainfall (RW, RW.40 and WW) conditions are referred to as “wet”. Hydric tensions were tracked at Mauguio using pairs of tensiometers in each block per condition at -30 and -60 cm depth, and pluviometers. These were always placed under the same hybrid to capture the field effect. Both tension and rainfall quantities were tracked daily (**Figures 1A, B**) to determine the irrigation needs.

Each condition at Mauguio was sown in three blocks of two lines per hybrid per condition, with all conditions separated by a 20-meter border to avoid accidental irrigation of deficit conditions. Versailles was sown in the same way. Blocks were randomised within each condition in incomplete Latin squares.

### 2.2 Agronomic analysis

Several key agronomic variables were selected for measurement per block and variety for the field trials. Male (when half of the given plants contained 2/3 open pollen pods on the tassels) and female (when half of the given plants contained visible silks) flowering was first recorded to determine the anthesis-silking interval (ASI). Plant height was also measured at the silage stage and averaged per block and variety by harvesting two homogeneous plants per line at ground level, giving a total of four plants per block and variety and condition. Furthermore, yield was estimated by grinding the same plants used for height measurements to determine the total aboveground wet biomass per hybrid and block. Plants were weighed using a precision industrial scale. After grinding in a Viking GE 355, a representative sample of 300 to 400 g was dried in a forced-air oven at 55°C for 72 h and grounded with a hammer mill (1 mm grid) for further use as biochemical samples and for determination of the percentage of DM.

### 2.3 Biochemical analysis

All samples were analysed for DM digestibility values as described in Lopez-Marnet et al., 2021. The most representative of the three available blocks for the five conditions was selected after principal component analysis of agronomic, DM digestibility, and NIRS values. CW residue (CWR) was extracted by the Soxhlet water/ethanol method (Effland, 1977), which allowed determination of calculated CW digestibility (Argillier et al., 1998) as follows: calculated CW digestibility = (100*(DM digestibility-(100-CWR))/CWR). The same method used to measure DM digestibility was repeated on the CWR samples to obtain measured CW digestibility values. Lignin content was measured through acetyl bromide (ABL) dosing using a method adapted from Fukushima and Hatfield (2001). The digestible yield was estimated by calculating the amount of digestible tons of DM/ha as follows: digestible yield = yield * (DM digestibility/100).

### 2.4 Construction of homegrown NIRS predictive equations on whole plant samples

We developed NIRS predictive equations to predict CW content, lignin content in CW, and DM and CW digestibilities of whole-plant forage maize samples. Calibration samples (218 in total) were selected from whole-plant samples of modern S0-S1 hybrids harvested at silage stage in 2018, 2019 and 2020 in different areas in northern France representative of the forage maize growing region. Biochemical quantifications were performed on the calibration samples following the same biochemical protocols as described above. Simultaneously, NIRS spectra were acquired on these 218 samples using a ThermoFisher Antaris II. Among them, 168 were used to generate the predictive equations, and 50 samples were used for external validation to assess the quality of these emerging equations (**Table 1**).

**Table 1:** DM and CW-related features measured in the laboratory, with their associated NIRS prediction equation features for the IJPB equation.

Trait	IJPB NIRS Designation	Units	Calibration		Validation		SEP/SECV^2^
			*n*	*r* ^1^	*n*	*r* ^1^	
DM digestibility	IJPB predicted DM digestibility	%DM	168	0.805	50	0.799	1.71
Calculated CW digestibility	IJPB predicted calculated CW digestibility	%CWR	168	0.812	50	0.877	3.11
Measured CW digestibility	IJPB predicted measured CW digestibility	%CWR	168	0.781	50	0.722	2.13
Lignin content	IJPB predicted lignin content	%CWR	168	0.593	50	0.319	0.64
Van Soest lignin content	IJPB predicted Van Soest lignin content	%NDF	155	0.700	0	0.597	0.22

**^1^** Calibration and validation r calculated via Pearsson; ^2^SEP, standard error of prediction; SECV, standard error of cross-validation.

### 2.5 NIRS predictions with two set of NIRS predictive equations

Predictions were made using two distinct NIRS equation systems, one developed by the CRA-W (Dardenne et al., 1993) and the other developed by the INRAE IJPB in Versailles and presented above, hereafter referred to as the “CRA-W equation” (abbreviated “CRA-W” in variable names) and “IJPB equation” (abbreviated “IJPB” in variable names), respectively. Both the CRA-W and IJPB equations often predict the same variables but with some special features (**Supplementary Table 2**), mainly due to CRA-W’s use of the Van Soest chain (Dardenne et al., 1993), which leads to a different quantification of CW as NDF and of the lignin content as Van Soest lignin content. The DM and calculated CW digestibility quantification are completely comparable between the two set of equations. On the other hand, the estimation of the measured CW digestibility is an important specificity of the NIRS equations developed at IJPB.

### 2.6 Statistical analysis

Data were statistically analysed using R 4.2.1 with the Rstudio interface (R Core Team, 2022; R Studio Team, 2022) and the Expé-R interface (ARVALIS, 2022a). Data were averaged across the three blocks for each variety per condition when possible. All data management was performed using tidyverse (Wickham et al., 2022). Data normality was checked using the Shapiro-Wilk test and residual dispersion analysis. ANOVA analysis was generally performed using a linear model that accounted for variety and condition. In cases where a column or row effect was detected, a linear model accounting for the relevant cofactor was implemented as follow:


$$Y_{ijkl}=\;µ\;+\;V_{i}+\;C_{j}+\;{\left(VC\right)}_{ij}+\;c_{jk}+\;r_{l}+\;E_{ijkl}$$

where *Y*_ijkl_ is the value for the given trait of the *i*th Variety in the *j*th Condition localized in the *k*th column and the *l*th row in the field. In this mode, µ is the intercept. An F test for interactions was performed to determine whether the additive model could be retained as previously described (Virlouvet et al., 2011). The significance levels for ANOVA, Tukey, and Pearson analyses were always set at 5%, unless otherwise stated. All correlations presented are first reported with the linear R^2^ value, as well as the significance value of the Pearson correlation. Residual standard deviations (RSD) were also calculated for most parameters.

Bar plots and dot plots were generated using the ggplot library (Wickham, 2016). When sufficient data points were available, error bars were added using the standard error values of each variable. Linear correlations were calculated using ggpubr (Kassambara, 2020), and Pearson correlations with their associated p-values were calculated using the ggcorr and corrplot packages (Kassambara, 2022; Wei and Simko, 2021).

## 3 Results

### 3.1 Water stress impacts on key agronomic traits

To quantify the effects of different water conditions on 13 hybrids representative of the French silage hybrids market, we evaluated three important agronomic traits (ASI, plant height, and DM yield) at three different water stress levels (no, moderate, and severe water stress, denoted WW, WD1, and WD, respectively) at Mauguio compared with rainy weather (denoted RW and RW.40) at Versailles (**Figures 1A–C** and **Supplementary Figure 1**). Although there were two harvest stages at Versailles, both had the same agronomic values except for yield, so we considered only the RW condition for the other traits.

First, we found a significant interaction between variety and condition for ASI (**Figure 1D** and **Supplementary Table 3**). The four conditions were divided into three distinctly different homogeneous groups from highest to lowest mean interval length: WD (2.33 days), WD1 with WW (0.87 and 0.74 days, respectively), and RW (-1.23 days) (**Supplementary Table 4**). The different varieties were also divided into three distinct categories (**Supplementary Table 4**): those with a longer interval only under the WD condition (varieties 4 and 11), those comparable under all Mauguio conditions but differing at Versailles (varieties 2, 5, 6, and 13), and the last final and largest group with much more mixed groups with a progressive decrease in ASI from the most to the least stressed (varieties 1, 3, 7, 8, 9, 10, and 12).

We also found a visible effect of the different irrigation conditions on plant height (**Figures 1C, E**). On average, plants reached 301 cm under the RW condition, lost 37 cm (264 cm) when switching to the WW condition, then another 53 cm (211 cm) under the WD1 condition, and, finally, another 42 cm (168 cm) under the WD condition. The ANOVA analysis revealed a significant interaction between variety and condition for these results (**Supplementary Table 3**), with each condition forming a distinct group that differed significantly in the order of their average height (**Supplementary Table 5**). Most varieties followed this order exactly with exception of variety 13, which did not differ in height between the WW and RW conditions.

Under the RW and WW conditions, yield was consistently similar across the different varieties, with corresponding averages at 16.69 and 17.36 tons of DM/ha (**Figure 1F**). The later harvest at 40% DM increased this yield to 18.38 tons of DM/ha. The WD condition reduced this yield to an average of 10.2 tons of DM/ha, partially restored under the WD1 condition by increasing to 14.29 tons of DM/ha. Significant variety and condition effects were found, but the interaction between variety and condition was not significant (**Supplementary Table 3**). It is worth noting that no difference was found between RW.40 and WW conditions and between WW and RW conditions (**Supplementary Table 6**).

### 3.2 Water stress significantly increases digestibility while decreasing yield, while a higher percentage of DM content at harvest has no effect on digestibility

**Figure 2:**
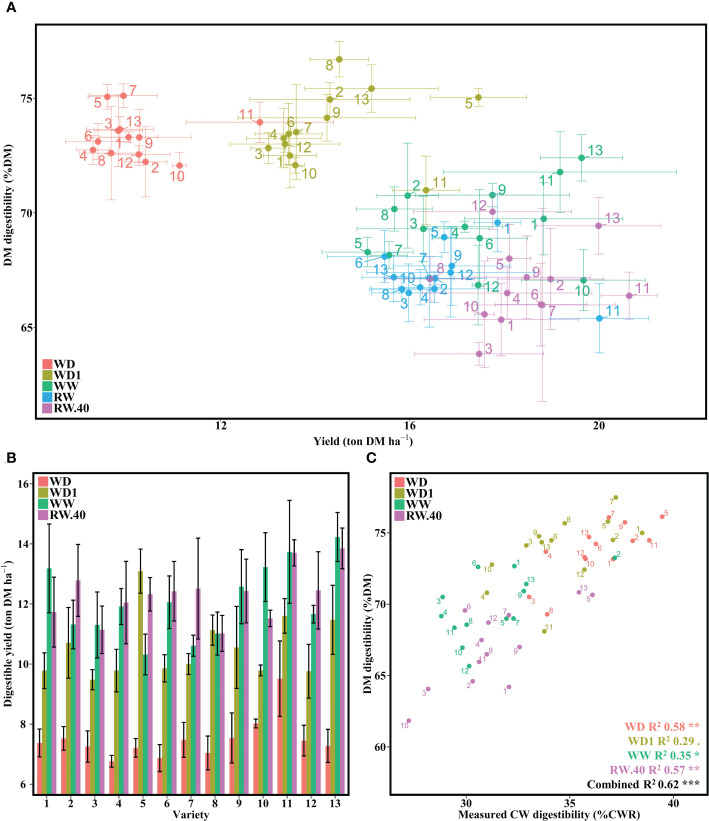
Effect of water deficit on DM digestibility, its combination with yield, and its relationship to measured CW digestibility. **(A)** DM digestibility to yield per variety under the five conditions. **(B)** Digestible tons of DM per ha averaged per condition for each variety under four conditions. **(C)** Relationship between laboratory measured DM digestibility and measured CW digestibility on a single representative block. Bars represent means and error bars represent standard error margins. Linear equation values are proceeded by Pearson correlation significance symbols: *P*<0.1:.; *P*<0.05: *; *P*<0.01: **; *P*<0.001: ***.

Because the criterion for quality of French forage maize is UFL, which is mainly determined by DM digestibility, we measured DM digestibility for all blocks for each variety under all conditions (n = 195) (**Figure 2A**). We found a strong condition effect alongside a smaller variety effect on DM digestibility (**Supplementary Table 3**). We also identified a total of three Tukey groups for the condition effect (**Supplementary Table 7**) in order from most to least digestible: (1) both water deficit conditions with nearly identical DM digestibility (mean DM digestibility for WD 73.33%DM and for WD1 73.68%DM), (2) WW alone (69.5%DM), and (3) both the RW (67.32%DM) and RW.40 (66.81%DM) conditions. Furthermore, there was a combined decrease in yield (average decrease of 7 points) associated with an average increase in DM digestibility of 4 points under the WD condition, while yield increased by more than 3 points under the WD1 condition, while the three wet conditions all appeared to be less digestible but more productive (**Figure 2A**). Some varietal effect was also found: genotype 5, for example, gained 8 points in DM digestibility and 3 points in yield in WD1 compared to WW. In contrast, variety 13 gained 3 points in DM digestibility, but lost 5 points in yield. A decrease in variability for both factors was also observed in WD condition compared to WW. This variability was partially restored in WD1, which was able to combine the good digestibility of WD with a higher yield.

Determination of digestible yield suggested a varietal effect, with certain varieties showing similar digestible yield under the WD1 condition as under the wet conditions (**Figure 2B**). As with the yield data, no interaction between variety and condition was found, although there was a significant effect for both separate factors (**Supplementary Table 3**). It is noteworthy that no significant difference was found between conditions WW and RW.40 (**Supplementary Table 8**).

To begin assessing the impact of CW on digestible yield, we retained a block, determined to be representative of the other two, to assess measured CW digestibility. We first observed an increase in CW digestibility under stress conditions, increasing from an average of 31.31 in WW to 34.63 in WD1 to 36.34 in WD (**Figure 2C**). No major difference was found between the various wet conditions. When compared to the corresponding DM digestibility values, we found significant correlation across all five conditions (*P <*0.1), with R^2^ values ranging from 29 to 57% depending on the condition. The decrease in variability with increasing water stress observed in **Figure 2A** also appeared to be lost for measured CW digestibility, with values evenly distributed across conditions.

### 3.3 NIRS predictive equations accurately measure both DM and CW digestibilities

While the biochemical results mentioned above are precise and repeatable, they lack the necessary throughput needed for selection programs and quality observations. Therefore, although certain protocols have achieved “high-throughput” status, NIRS still remains the preferred method to measure various DM- and CW-related parameters and constitutions, although some work is still needed to affine these predictive equations. Consequently, we compared the predictive power of an older and extremely well-tested CRA-W equation with our own emerging IJPB equation for biochemical values measured under four of the five original conditions (**Table 1** and **Supplementary Table 7**). The lack of a significant difference between the two Versailles conditions (**Supplementary Table 7**) prompted us to continue all further analyses, after removing the RW condition while retaining the RW.40 condition, which was more similar to the conditions found at Mauguio in terms of maturity stage.

**Figure 3:**
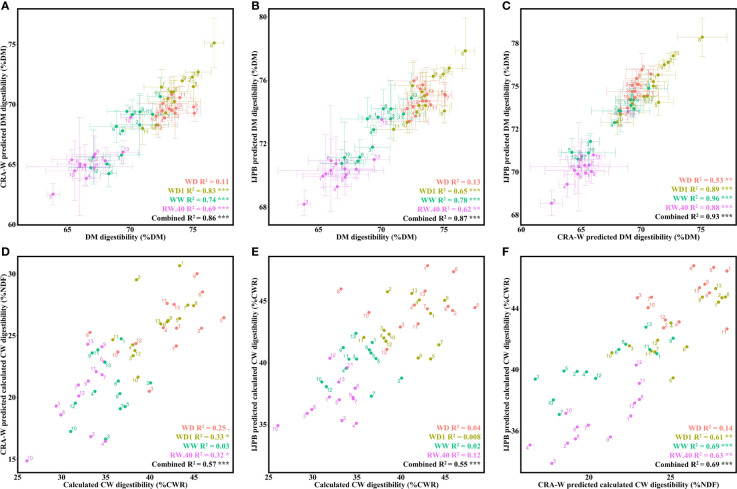
Prediction accuracy of CRA-W and IJPB NIRS predictive equations for both DM and CW digestibilities under different hydric conditions. **(A)** Relationship between DM digestibility predicted by CRA-W and DM digestibility measured in the laboratory. **(B)** Relationship between IJPB-predicted and laboratory-measured DM digestibility. **(C)** Relationship between the two predicted means from the IJPB and CRA-W equations for DM digestibility. **(D)** Relationship between laboratory-calculated CW digestibility and CRA-W-predicted CW digestibility. **(E)** Relationship between laboratory calculated mean CW digestibility and IJPB-predicted calculated CW digestibility. **(F)** Relationship between IJPB-predicted calculated CW digestibility and CRA-W-predicted CW digestibility. The dots denote the ratio values for the individuals, unless they are given as mean values, in which case they represent the mean values of the 3 blocks. Linear equation values are proceeded by Pearson correlation significance symbols: *P*<0.1:.; *P*<0.05: *; *P*<0.01: **; *P*<0.001: ***. The error bars represent the standard error margins.

Both equations were found to predict the biochemical values of DM digestibility with high accuracy under most conditions (most R^2^ values ranged from 62 to 83% per condition, and overall tested environments combined produced significant correlations with values of 86% and 87% for CRA-W and IJPB, respectively) (**Figures 3A, B**). It is worth noting that no equation managed to predict DM digestibility values in the WD condition. It was also found that both equations correlate strongly with each other, with a slight drop in accuracy in the WD condition (**Figure 3C**).

Regarding the calculated CW digestibility values, we first noticed a decrease in the prediction accuracy: most R^2^ values were between 32 and 33% (with significantly correlated condition-combined values at 57% and 56% for CRA-W and IJPB, respectively) (**Figures 3D, E**). The CRA-W equation appears to be more precise for the calculated CW digestibility, providing significant correlations within each condition, while the correlations produced by the IJPB equations in WD, WD1, WW and RW.40 were insignificant. Similarly, to the DM digestibility, both equations correlated significantly with each other, except for the WD condition (**Figure 3F**).

**Figure 4:**
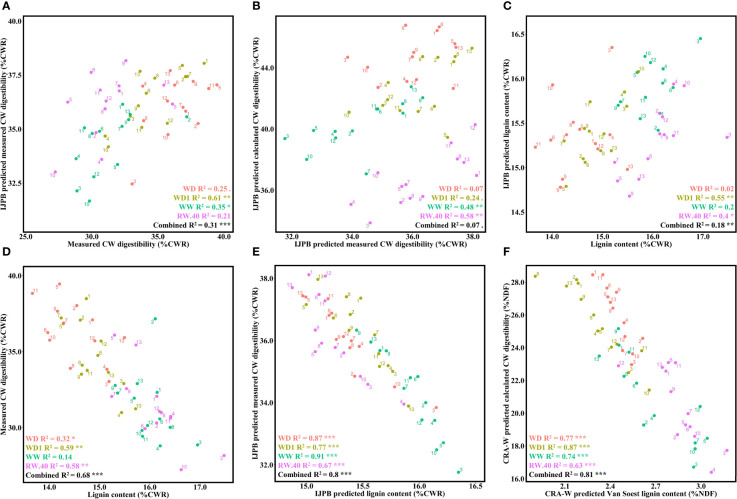
Lignin content and its role in CW digestibility and prediction of the relationship. **(A)** Relationship between laboratory-measured CW digestibility and IJPB-predicted measured CW digestibility. **(B)** Relationship between mean IJPB-predicted values for measured CW digestibility and IJPB-predicted calculated CW digestibility. **(C)** Relationship between laboratory-measured lignin content and IJPB-predicted lignin content. **(D)** Relationship between laboratory-measured lignin content and laboratory-measured CW digestibility on a representative block. **(E)** Relationship between mean IJPB-predicted values for lignin content and IJPB-predicted measured CW digestibility. **(F)** Relationship between CRA-W-predicted lignin content and CRA-W-predicted calculated CW digestibility. The dots denote the ratio values for the individuals, unless they are given as mean values, in which case they represent the mean values of the 3 blocks. Linear equation values are proceeded by Pearson correlation significance symbols: *P*<0.1:.; *P*<0.05: *; *P*<0.01: **; *P*<0.001: ***. The error bars represent the standard error.

In parallel with the calculated CW digestibility prediction equation, we developed a predictive equation for measured CW digestibility. This measurement provides a much more accurate representation of the true digestible fraction of CW and is therefore a better criterion for improving DM digestibility. While the CRA-W equation did not predict measured CW digestibility, the IJPB equation did. Depending on the condition, the values predicted from this measurement correlated relatively well with the biochemical values (**Figure 4A**). While no significant correlation was found for the WD and RW.40 conditions, the correlation for the other two conditions typically ranged from 35 to 61% (condition-combined at 33%). One might assume that the two predicted versions of the calculated and measured CW digestibility would correlate relatively well, but we found that this was not the case: correlation values between the two measurements varied significantly depending on the condition considered (**Figure 4B**). No significant correlation was found under the WD condition, and the correlation values ranged from 24 to 58% depending on the condition considered, but dropped to **7**% when all conditions were considered.

### 3.4 Heading deeper into the CW seems to reduce NIRS accurateness

To better understand and thus improve the variations in digestibility of DM and CW, it is necessary to know the composition of this CW and, in particular, its lignin content. Consequently, high-throughput analytical methods are required to determine the lignin content. Therefore, we measured the total lignin content using the ABL method on samples from the same block used to measure CW digestibility. The IJPB equation used to predict lignin content was not very efficient and needs to be improved. In fact, significant correlations were found for only two of the four conditions in WD1 and RW.40, with a combined correlation of 18% (**Figure 4C**).

The effects of lignin content on measured CW digestibility were then quantified (**Figure 4D**). Both traits were significantly correlated in all conditions, except the WW condition, where correlation values ranged from 14 to 59%, with an overall value of 68%. This correlation was then determined with NIRS-only data using the CRA-W and IJPB equations. Although the CRA-W equation does not accurately predict the same variables, the measured CW digestibility and lignin content were approximated to the calculated CW digestibility and Van Soest lignin content, respectively, with the latter allowing determination of the more condensed lignin fraction (Zhang et al., 2011). Regardless of these changes, both equations (IJPB, **Figure 4E**; CRA-W, **Figure 4F**) find highly significant correlations between CW digestibility and lignin content, regardless of conditions, yielding values much stronger than those found in the biochemical results.

### 3.5 Correlations between traits reflect genetic relationships when separate conditions are considered

**Figure 5:**
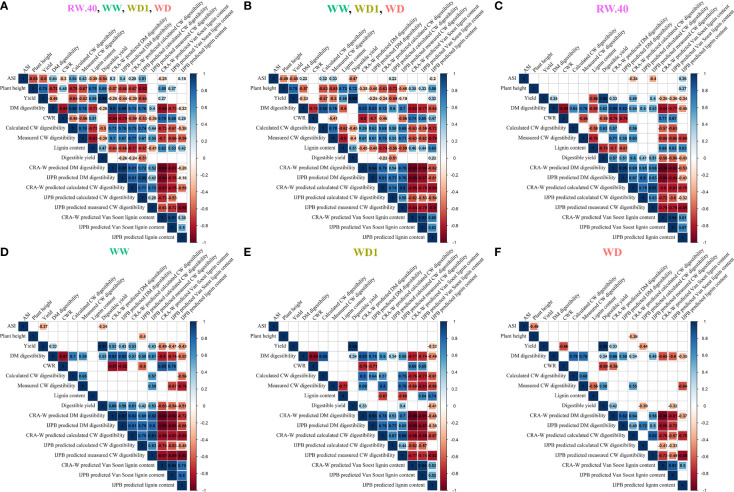
Correlation matrices for the main considered agronomic, biochemical and NIRS-predicted variables observed during the study at the different field trial locations and under different conditions. Correlation matrices for all variables considered, including some used for computational purpose only, separately for all conditions combined in **(A)**, for all Mauguio site conditions in **(B)**, for the main-Versailles RW.40 condition retained for the study in **(C)**, and then separately for the three Mauguio conditions of WW in **(D)**, WD1 in **(E)**, and WD in **(F)**. Pearson correlations with correlation values in black on the matrix, significance threshold *P* = 5%. Empty squares indicate non-significant correlations.

To visually and statistically quantify the effects of the different water conditions on the relationships between the different agronomic, biochemical, and NIRS-predicted variables examined in this study, we plotted all variables in a correlation matrix for all combined conditions (**Figure 5A**), the three contrasting conditions at Mauguio (**Figure 5B**), and each condition separately: RW.40 (**Figure 5C**), WW (**Figure 5D**), WD1 (**Figure 5E**), and WD (**Figure 5F**). Overall, we observed many more significant correlations (positive or negative) when the four (**Figure 5A**) or three (**Figure 5B**) environmental conditions were included in the analysis than when the environmental conditions were considered separately (**Figure 5C** through **Figure 5F**) and only genetic variation was included. It is also worth noting that a negative correlation between DM digestibility and yield was observed both in the matrix of all conditions (R = -0.46) and in the three contrasting conditions in Mauguio (R = -0.33). Importantly, this correlation became positive or absent when the environmental conditions were considered separately. Moreover, the correlation between measured CW digestibility and lignin content was always extremely negative, regardless of the conditions, except for the WW condition. It should also be noted that these correlations were strongly exacerbated when values were predicted by NIRS (WD -0.88 versus -0.56, WD1 -0.82 versus -0.77, WW -0.92 versus none, and RW.40 -0.85 versus -0.76).

## 4 Discussion

While climate predictions generally point to a drier future, it is not yet known with full certainty exactly how dry it will be. Chaotic episodes seem inevitable, and crop conditions are therefore difficult to predict each year. It is therefore important to understand the effects of environmental conditions on yield and digestibility of forage maize. For this reason, our study relied on two locations and two water stress levels, one moderate and the other severe, which in combination represent four very different environmental conditions. During the growing season, precision instrumentation and daily monitoring in the field, where several factors, including soil heterogeneity, are not typical of greenhouse conditions and, consequently, difficult to replicate (Li et al., 2022), allowed the two water stress conditions to remain distinctly different and finely tuned, resulting in contrasting agronomic and biochemical outcomes across the four environmental conditions. Therefore, we used them to examine the effects of both stress environment per se and stress intensity on this group of traits.

### 4.1 Daily monitoring of moderate water stress was key to identifying varieties with different agronomic responses to water stress

It was found that the ASI properties depend on the conditions and the genotype considered. It is worth noting that the ASI was not the same in the two wet conditions: it averaged -1 day in RW, while it averaged +1 day in WW. These inverse shifts in male and female flowering could be due to thermal rather than hydric constraints, since water stress is known to increase ASI (Claassen and Shaw, 1970). However, this increase was not observed under moderate water stress. Thus, our results suggest that a threshold level of water stress must be exceeded to affect flowering, consistent with previous findings showing that the proportion of yield variation explained by individual traits is small at intermediate levels of water deficit stress (Cooper and Messina, 2023). This threshold for ASI also appears to be dependent on variety, with some individuals not being affected by water stress at all. Genotype 6 is atypical as it seemed to synchronize its flowering better under stress conditions, while it had a strongly negative ASI under irrigated conditions in northern France. It is the opposite of the majority of the hybrids studied in our work. Genotypes 3 and 4 are the perfect counterexamples with a very low ASI under wet conditions and a high ASI under intense water stress condition. Lowering ASI has long been a selection objective to reduce the risk of kernel formation failure which can lead to loss of grain yield (Robins and Domingo, 1953). Despite selection efforts, there are still differences in ASI response to water stress among hybrids, and selection efforts to stabilize this key agronomic parameter are still needed in the context of climate change.

Both plant height and yield decreased under water stress conditions in our study, and did so incrementally depending on the severity of the stress. In addition to incremental yield decline under each condition, results were also less variable as a function of stress severity. The WD1 condition allowed some yield recovery depending on the variety, with variety 11, for example, producing a sufficient yield comparable to WW results. The effects of water stress on plant height are well studied, and the results found in our study are consistent with those found in the literature for miscanthus and maize (Emerson et al., 2014; Néné-bi et al., 2022), and also for sorghum and sugarcane (dos Santos et al., 2015; Perrier et al., 2017). We hypothesize that one of the reasons for the reduction in total plant height under water stress is the reduction in internode length, as noted by Sah et al., 2020. While plant height is most likely responsible for a large part of the yield change in water stressed conditions, other factors can also be considered. Grain yield, which has been replaced by UFL in the official selection criteria for forage maize, accounts for 30 and 52% of plant biomass and is also known to be adversely affected under drought conditions (Ferreira and Brown, 2016). The greater effect on ASI in WD than in WD1, as well as the maintenance of a severe stress until silage harvest and thus during the grain-filling period, could explain the lower effects of moderate stress on yield. The increase in variability under WD1 conditions was key to this study. Indeed, while yield results under WD were nearly identical with little to no variation, the slight increase in irrigation under WD1 increased variability in results and allowed us to identify varieties with different responses to moderate stress, including potential varieties with novel responses such as those observed in varieties 5 and 11.

### 4.2 High-throughput NIRS predictive equations reliably predict basic forage quality characteristics, such as digestibility, but significantly exaggerate correlations between biochemical parameters

NIRS predictive equations have been developed for 50 years to predict forage quality (Norris et al., 1976) and are available for maize harvested at the silage stage to evaluate its digestibility and composition (Dardenne et al., 1993). Several authors emphasize that CW-related traits in maize stover can be accurately predicted. For example, DM and CW digestibilities are successfully predicted using several published equations (Lübberstedt et al., 1997; Riboulet et al., 2008; Jung and Phillips, 2010; Virlouvet et al., 2019), as are traits related to CW composition (Dardenne et al., 1993; Lorenz et al., 2009; Jung and Phillips, 2010; Virlouvet et al., 2019). The DM and CW digestibilities equations developed in this study also showed a good r of validation for the above-mentioned traits. In contrast, the r of validation for both ABL- and Van Soest-based lignin content was not very high and, importantly, much lower than that we developed on whole plants without ears (Virlouvet et al., 2019). This is most likely due to the lower variation in lignin content in modern French forage maize hybrids compared to the variations observed within maize inbred lines found in Virlouvet et al. (2019).

The predictive equations developed by CRA-W (Dardenne et al., 1993) for maize whole-plant samples have long been routinely used in seed company breeding programs, and we therefore decided to compare the predictive values of our young IJPB equations with those of CRA-W. Both NIRS predictive equations consistently predicted DM digestibility accurately. The IJPB equation also efficiently predicted measured CW digestibility. These predictions were affected by the different environmental conditions, and neither equation was able to correctly predict the values for the WD condition, where the variation in CW digestibility is greatly reduced. When predicting the same trait, both equations predicted values were also highly correlated with each other. It is worth noting that calculated CW digestibility was always poorly predicted by both equations despite the overall good prediction quality of these equations. Prediction accuracy increased for measured CW digestibility, which, interestingly, was itself poorly correlated with calculated CW digestibility. Although younger and a much smaller calibration pool, we found that the IJPB predictions are therefore comparable to the ones from CRA-W equation in all aspects considered in our study. In general, it is considered that the calibration set must be homogeneous. In the case of a large sample as discussed by Bastianelli et al. (2019), bases with large variability or mixtures make interesting the use of so-called “local” regression techniques (Shenk et al., 1997), which consist in searching and selecting in the samples a calibration set whose spectra are close to the sample to be predicted, and building a non permanent calibration model with this specific subset. Thus, it is certainly necessary to think differently when constructing predictive equations to accurately characterize the variability in CW composition and digestibility.

While the predictive accuracy of individual traits is useful for selection purposes and for identification of biochemical targets, the correlation between these traits is often more informative. Biochemically, we found that lignin content explains between 14 and 58% of the measured variation in CW-digestibility depending on the given environmental conditions. When we attempted to replicate this correlation with purely predicted values from the CRA-W and IJPB equations, the correlations were always highly significant and far stronger than those found biochemically. This overestimation of correlations prevents the use of predictions to find targets for digestibility improvement. For example, in WW, although the correlation between CW digestibility and lignin content is not significant when the values are estimated biochemically, this correlation reaches r² values of 0.91 and 0.74 for the values predicted by the IJPB and CRA-W equations, respectively. For the CRA-W equation, we originally assumed that this excess correlation was due to the size of the calibration data set. Our hypothesis was that in such a large calibration pool, there were likely two groups of samples for lignin content, both stressed and non-stressed samples or mature and non-mature samples, which produced a direct linear equation between the two groups. While this could explain the strong correlations in the CRA-W equation, this idea loses ground when we consider that this cannot apply to the IJPB equation, which also has strong correlations but does not include samples under stressed conditions or with different degrees of maturity. Including samples in an equation that come from a range of different stress levels, such as under WD and WD1 conditions, could potentially mitigate this problem. Another alternative, albeit a complex one, would be to create NIRS equations for different types of environments. By using a calibration set consisting solely of samples from water stressed environments, we could improve the accuracy for these conditions, provided that the traits are variable. This would require determining the various conditions that could be specified with specific stress thresholds. These results are of particular interest because selection and quality assurance programs typically consider only such correlations for evaluations, CW digestibility to lignin content (or equivalent) being a common example.

### 4.3 Although both DM and CW digestibilities lose variability under water deficit, they are increased under this stress condition simultaneously with a decrease in lignin content

We found that both DM and measured CW digestibility gradually increased as a function of the severity of water stress. Average DM digestibility did not change between WD and WD1, but decreased significantly under the wet conditions. The Versailles results also showed that DM digestibility was identical under RW and RW.40 conditions. Assuming the silage is well conserved, this result is reassuring from a farmer’s perspective. Indeed, the current chaotic climate often makes it difficult to predict when silage will be harvested. However, we have shown here that a later harvest (40% DM content instead of 32-34%) does not reduce DM digestibility and thus the UFL value of the forage. Interestingly, our results also indicate that genotypic variability decreases under the most stressed conditions, almost halving the spread between RW.40 and WD conditions. The WD1 condition allowed recovery of this loss of variability, as in yield. This loss was also seen in measured CW digestibility, but to a lesser extent. Lignin content was similarly affected, with lower values detected in the WD and WD1 conditions, peaking in WW and RW.40. It was also found that measured CW digestibility and lignin content were significantly negatively correlated in three of the four conditions. However, biochemically, variations in lignin content explained at most 35% (WD1 condition) of the observed variations in CW digestibility. It is noteworthy that lignin content varied by only one point and that this small variation is difficult to detect.

These results are consistent with previous work on maize inbred lines where water stress was found to have similar effects on lignin content and digestibility (El Hage et al., 2018; Virlouvet et al., 2019; El Hage et al., 2021), as well as, results observed in other grasses (Emerson et al., 2014; Sanaullah et al., 2014; Perrier et al., 2017). What is striking about the results of maize hybrids compared to those of maize inbred lines is the low variation in lignin content independent of water conditions. In Zhang et al. (2011), we intentionally selected maize inbred lines with comparable lignin content and still had a 3-point of variation in lignin content in the CW. Modern maize hybrids have very similar lignin contents and have a range of variation of about 1 point, as we have shown here and by Baldy et al. (pers comm) and Lopez-Marnet et al. (2021). Lignin content has been constrained by selection of S0-S1 maize hybrids and has likely found an optimum that provides resistance to fungal attack and lodging, high yields and good digestibility. We believe that the lack of difference in digestibility between RW and RW.40 conditions is likely due to an increase in the proportion of digestible grains that offsets the reduction in leaf and stem digestibility (Khan et al., 2015). Again, the WD1 condition led to an increase in variability in results, as was observed for yield. This opens reassuring perspectives in the context of climate change. First, we have shown that in the case of a moderate but still significant and long stress for the growing season of maize, there is still genetic variability for yield and digestibility that can allow the selection of maize hybrids whose yields are maintained and whose digestibility is increased. This increase in digestibility while maintaining yields has been observed several times in the ARVALIS network (ARVALIS, 2022b) and in agriculture during the last hot and dry summers in France. Moreover, we found significant correlations between measured CW digestibility and lignin content, although lignin content has long been counter-selected in selection programs for forage maize. While lignin content in inbred lines of maize in contrasting environments typically varies between 11 and 21% (El Hage et al., 2018), variability in lignin content in modern maize hybrids always studied in contrasting environments, ranged from 13.6 to 17.5%. This narrow range severely limits the potential for improving this trait. However, to improve the UFL value of forage maize, we need to increase DM digestibility and, consequently, CW digestibility. New targets or combinations of targets need to be found. These could be new biochemical traits such as *p*-coumaric acids or histological traits (Méchin et al., 2005; Zhang et al., 2011; El Hage et al., 2021; Zhang, 2021; Lopez-Marnet et al., 2022) to study the localization of lignified tissue by FASGA staining of internode cross section.

### 4.4 Less productive but more digestible: Moderate stress could help compensate for losses due to water deficiency

Yield values were linked to digestibility data from DM to obtain a quantification of “digestible yield” in tons of digestible DM/ha, which allows us to better link these two criteria. The highest digestible yield values were typically found in the WW and RW.40 conditions, followed by values from WD1 and then WD. Certain varieties under the WD1 condition managed to raise yield to levels achieved under irrigated or rainfed conditions, such as varieties 5 and 8. We also found that while yield was negatively correlated to DM digestibility when all conditions were analysed together, this negative correlation disappeared once each condition was considered separately. The lack of antagonism between these two key traits in selection is important, especially since selection for yield has led to a decline in digestibility (Barrière, 2000; Surault et al., 2005; Baldy et al., 2017).

The WD1 condition was irrigated 37% more than the WD condition, and 56% less than the WW condition. Importantly, an average of four tons DM/ha of biomass yield or three tons of digestible DM/ha was recovered, allowing the condition to recover up to 82% of the biomass and 87% of the digestible yield of the WW condition. These results would likely lead to even more dramatic differences in irrigation in commercially irrigated maize fields, where the decision to irrigate is rarely supported by precision tools such as those used in our study. Only 7.6% of farmers irrigating in Alabama (USA) used tension meters in 2018 (USDA-NASS, 2019). In France, this number is likely even lower because forage maize acres are managed by smaller farms with less access to the training and materials needed to monitor soil water tension. In trials comparing conventional and precision irrigation, full yield recovery was possible while water use for grain was reduced by 25% (Bondesan et al., 2023). Clearly, even a relatively small reduction in stress can easily contribute to recovery of productivity.

Out of the 13 varieties evaluated in our study, variety 11 proved to be the best performer under the various conditions. It will be of interest to study the biochemical and histological differences between this variety and the others to determine what allows this different response. Similarly, although to a somewhat lesser extent, varieties 2, 5 and 9 are also of interest in this section, primarily because of their ability to maintain productivity. These varieties, all performed well under both moderate stress and irrigation conditions, likely contain important potential selection targets to identify.

## 5 Conclusion

Establishment of specific water stress levels in the field allowed us to understand how water stress and its intensity affect yield and digestibility of modern forage maize hybrids. Our data showed that forage yield gradually decreased as a function of water stress, while both DM and CW digestibilities increased regardless of water stress intensity, with decreased variability among tested varieties under severe water stress conditions. Overall, our work also showed that there was no antagonism between yield and digestibility under each environmental condition studied and that improvement in DM digestibility requires improvement in CW digestibility.

## Data availability statement

The raw data supporting the conclusions of this article will be made available by the authors, without undue reservation.

## Author contributions

SC, VM, AU and NM designed the study. The field trials were organised by SC, VM, together with MR and CM for Versailles, by FM, together with SR, SF and PS for Mauguio, and by NM, MR, VM and P-LL-M for the different areas in northern France used for calibration. M-PJ was responsible for both harvesting and planning logistics in the laboratory. OM, M-PJ, CD, MR, P-LL-M, VM and SC collected the samples. YG recorded the NIRS spectra and developed the IJPB NIRS equations. DM digestibility was performed by OM, SG and CD. CWR extraction and CW digestibility data were obtained by M-PJ, SG, and OM. Lignin content was measured by M-PJ. Data analysis was performed by OM, SC and VM. OM, SC and VM wrote the manuscript. All authors read and approved the submitted manuscript.

## Supplementary material

Supplementary Figure 1 Water balance sheet generated by the Irrélis irrigation advice software (ARVALIS – Institut du vegetal) for the Versailles field trial. The data shown were measured on variety 8 in the RW condition.
